# Wound healing in wild male baboons: Estimating healing time from wound size

**DOI:** 10.1371/journal.pone.0205017

**Published:** 2018-10-09

**Authors:** Haruka Taniguchi, Akiko Matsumoto-Oda

**Affiliations:** 1 Faculty of Global and Regional Studies, University of the Ryukyus, Nishihara, Okinawa, Japan; 2 Mpala Research Centre, Nanyuki, Kenya; 3 Graduate school of Tourism Sciences, University of the Ryukyus, Nishihara, Okinawa, Japan; University of Pisa, ITALY

## Abstract

Wound healing in animals is important to minimize the fitness costs of infection. Logically, a longer healing time is associated with higher risk of infection and higher energy loss. In wild mammals, wounds caused by aggressive intraspecific interactions can potentially have lethal repercussions. Clarifying wounding rate and healing time is therefore important for measuring the severity of the attacks. In addition, impact of secondary damage of wounds (e.g., accidental peeling off of scabs) on heeling time is unknown despite the risk of infection in wild mammals. In baboons, most male injuries have been reported to result from male to male fights. Here, we investigated the relationship between wound size and healing time in wild anubis baboons to clarify the healing cost of physical attacks including secondary damage of wounds. Observations were conducted daily between August 2016 and July 2017 in Kenya for seven adult male anubis baboons. The individual wound rate was one per month on average. In 16 cases, we were able to assess the number of days required for wound healing, and the median healing time was 13 d. Wound healing time was longer for larger wounds. When the scab was peeled off accidentally because of external factors, healing time became longer. One of the causes of scabs’ peeling off was baboons’ scab-picking behavior, and the behaviour was considered self-injurious behavior. However, its predicted healing cost might not be high. We concluded that wounds less than 800 mm^2^ (the largest observed in this study) in baboon males have little effect on survival. Our results suggest that lethal wounds by physical attacks rarely occur in male baboons, and that healing time and delay caused by secondary damages can be estimated by measuring wound area.

## Introduction

In animals, wound healing is important to minimize the fitness costs incurred by infection [1–5]. When cutaneous wounds fail to heal, animals can experience high risk of infection [2, 3]. Moreover, injuries can affect reproductive activities, increase predation risk, and decrease feeding opportunities in wild vertebrates such as lizards [6], bats [7], red deer [8, 9], and primates [10–12].

Wound healing is costly in terms of energy [13–15]. Logically, a longer healing time is associated with a higher risk of infection or suppuration, and higher energy loss to the healing process. Healing time is therefore an important indicator for measuring wound severity, particularly in the wild, where bacterial and viral infections are more likely to occur than under laboratory conditions [14]. A few studies measured healing time in the wild [10, 15–19]. Whereas small wild vertebrates (e.g., lizards [15] and bats [16, 19]) can be captured, subject to standardized wounds, and then returned to their natural habitat where wounds can be monitored by recapturing these animals, large- and medium-sized wild vertebrates cannot be subjected to standardized wounds, released, and then recaptured. Instead, researchers wait for natural wounds to occur in target animals and record qualitative observations of wound healing [10, 17, 18], and thus little is known on the relationships between wound area and healing time in large- and medium-sized wild animals.

Moreover, in wild animals, the effect of secondary damage to wounds (e.g., peeling scabs) on heeling time has not been investigated, despite their recognized risk of infection. When scabs are peeled off accidentally during the healing process, the risk of infection increases, and healing time might increase. Scabs are formed on the wound surface during the healing process [20], and peel off naturally at the end of epidermis regeneration. Although scabs have some functions, including provision of a barrier against foreign material, holding wound edges in approximation, facilitation of wound contraction, and minimization of fluid loss [21], they have been reported to slow epithelialization [22]. The influence of peeling of scabs accidentally on healing time is unknown in wild animals.

Wounds caused by aggressive intraspecific interactions can potentially have lethal repercussions in wild mammals [23, 24]. Adult males wound more often than other age/sex classes in several mammals (e.g., elephant seals [25], bottlenose dolphins [26] and primates [27, 28]), and most of these wounds have been reported to result from fights between males (e.g., muskox [29], deer [8, 9, 30], and primates [28, 31]). Escalation of every fight between males is disadvantageous in social mammals because they could drop their rank, and a third individual might benefit more than either contestant [23, 32, 33]. Wound costs of fights between males can be elucidated quantitatively by measuring wound healing time.

Male baboons, which are medium-sized mammals, are suitable subjects for determining the severity of wounds because several studies are available on their wounds in the wild [10, 17, 18, 28, 34]. In addition, most injuries in male anubis baboons (*Papio anubis*) have been reported to result from fights between males [28]. Moreover, mutual biting in fights between males is more common than non-mutual biting [34], and both winners and losers can be wounded during fights, although anubis baboons seem to avoid seriously wounding each other. However, it is unclear how much healing costs when male baboons sustain large injuries because catching medium-sized wild primates to assess healing time is difficult.

The present study aimed to determine the cost of wounds by measuring their healing time in wild anubis baboons, because assessing the consequences of physical attacks between males of this species is important. Previous studies in wild primates have adopted wound length as an indicator of wound size [10, 17]. However, because wounds spread in two dimensions, it may be insufficient to track one-dimensional length changes during wound healing. As so, we first constructed a “healing model” based on wound area to predict healing time (in days), and using this model we then elucidated the predicted delay in healing at the secondary damage of the wound. We predict that healing time is delayed when scabs were peeled off accidentally. The findings of the present study allow quantifying healing time, which might be used as a predictor of the cost of wounds, including the cost of secondary damage (e.g., peeling scabs).

## Methods

This research was approved the National Commission for Science, Technology, and Innovation of Kenya (NACOSTI/P/16/84320/12475).

### Definitions of terms

Cutaneous wounds: Wounds (cut, tear, and puncture) [10] that result in a complete breakage of the skin. Blood or scabs can be observed on the wound area. We did not include superficial wounds that result only in partial breakage of the skin [35].
Scab: Dark red or brown skin crust formed when serum leaks from a damaged area, mixes with pus and dead skin, and then clots.
Healing day: Cutaneous wounds were considered healed when there was no scab on the wound site, leaving only scars or re-epithelialized tissue [10, 17, 18]. The healing day was confirmed by checking the day on which the wound was healed and the day before the wound was healed. in order to know the accurate date when the scab naturally peeled off.
Accidental peeling off of scabs (APS): A scab formed, but peeled off early during the healing process, leading to the exposure of the red part of the wound; however, another scab formed subsequently, and healing resumed. We called a case that included an APS day "APS case". We did not include the case wherein scabs peeled off naturally once the wound had healed sufficiently, and no secondary scab was formed. We called a case that did not included any APS day "non-APS case".

### Study site, period and subjects

The study was conducted at the Mpala Research Centre, Laikipia District, Kenya (0°17'N, 36°53'E). We conducted field observations for 271 days, from August 19, 2016 to July 23, 2017. The anubis baboon (*P*. *anubis*) AI group, which had been habituated since 2011 by A.M.O. [36] was used as the subject group, and comprised 52–58 individuals, including infants, throughout the study period. Our study subjects were seven adult males. Their age-classes were judged based on apparent weight, size of canines and testicles, and immigration history. During the study period, due to immigration, the number of adult males in group AI changed from four to six individuals. We did not analyze the relationship between social rank and wound healing time because social rank among adult males changed during the study period.

### Data collection

**Fig 1:**
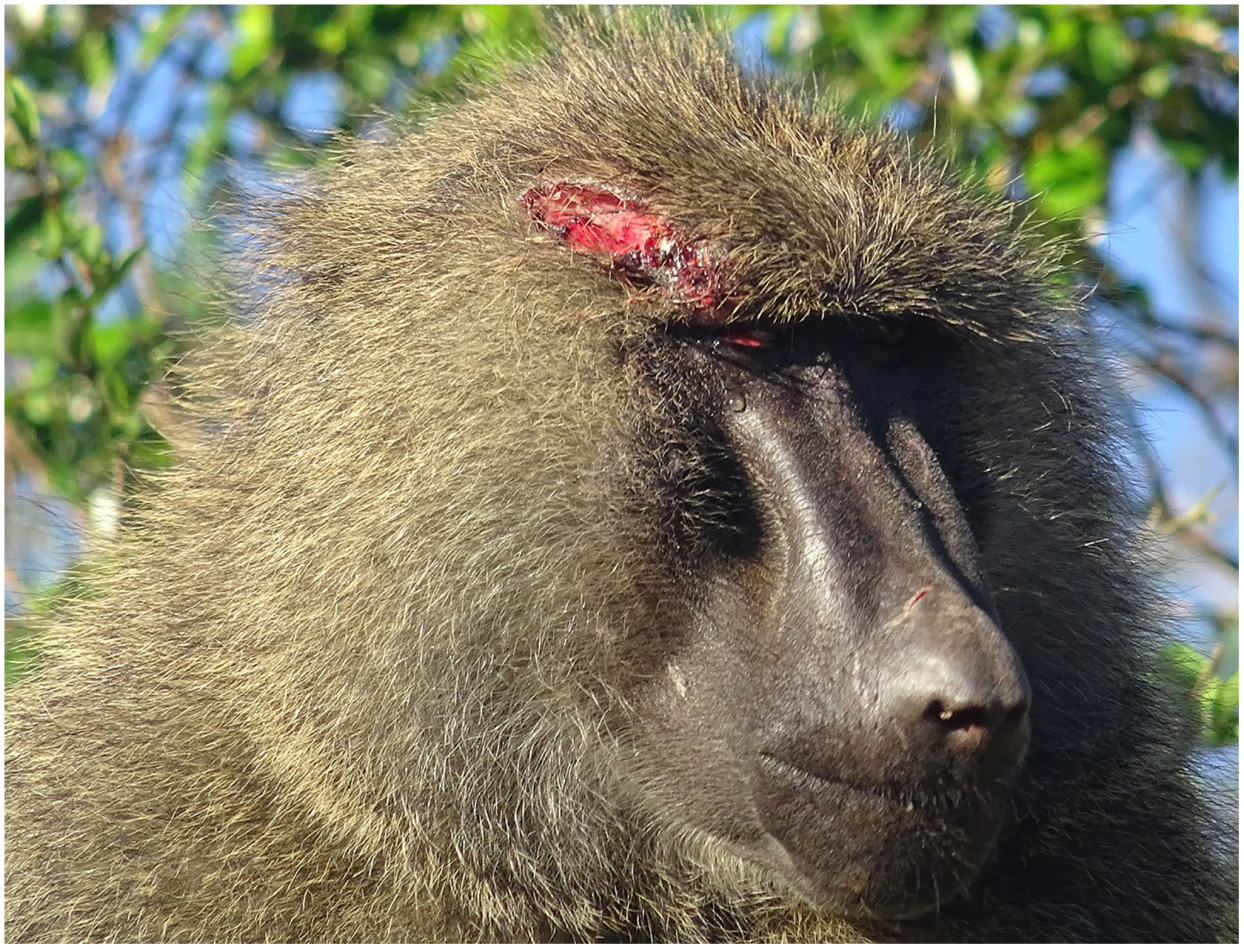
Examples of cutaneous wounds of the anubis baboon male.

On each day of the observation period, we checked whether all adult males in our subject group were wounded. When subjects experienced cutaneous wounds (e.g., Fig 1), we took periodic photographs or videos of the wounds until they had healed. When wounds were concealed beneath hair, measurement of the wound area was difficult. In the 16 cases included in our analysis (S1 and S2 Tables), the average observation interval for wounds (observation date to previous observation date) was 1.34 d (range: 1.0–6.0 d). When the photographs that were not sufficiently clear to measure the wound area were excluded from the analysis, the average measurement interval for the wounds was 1.81 d (range: 1.0–6.0 d).

### Photography-based body size and wound measurements

**Fig 2:**
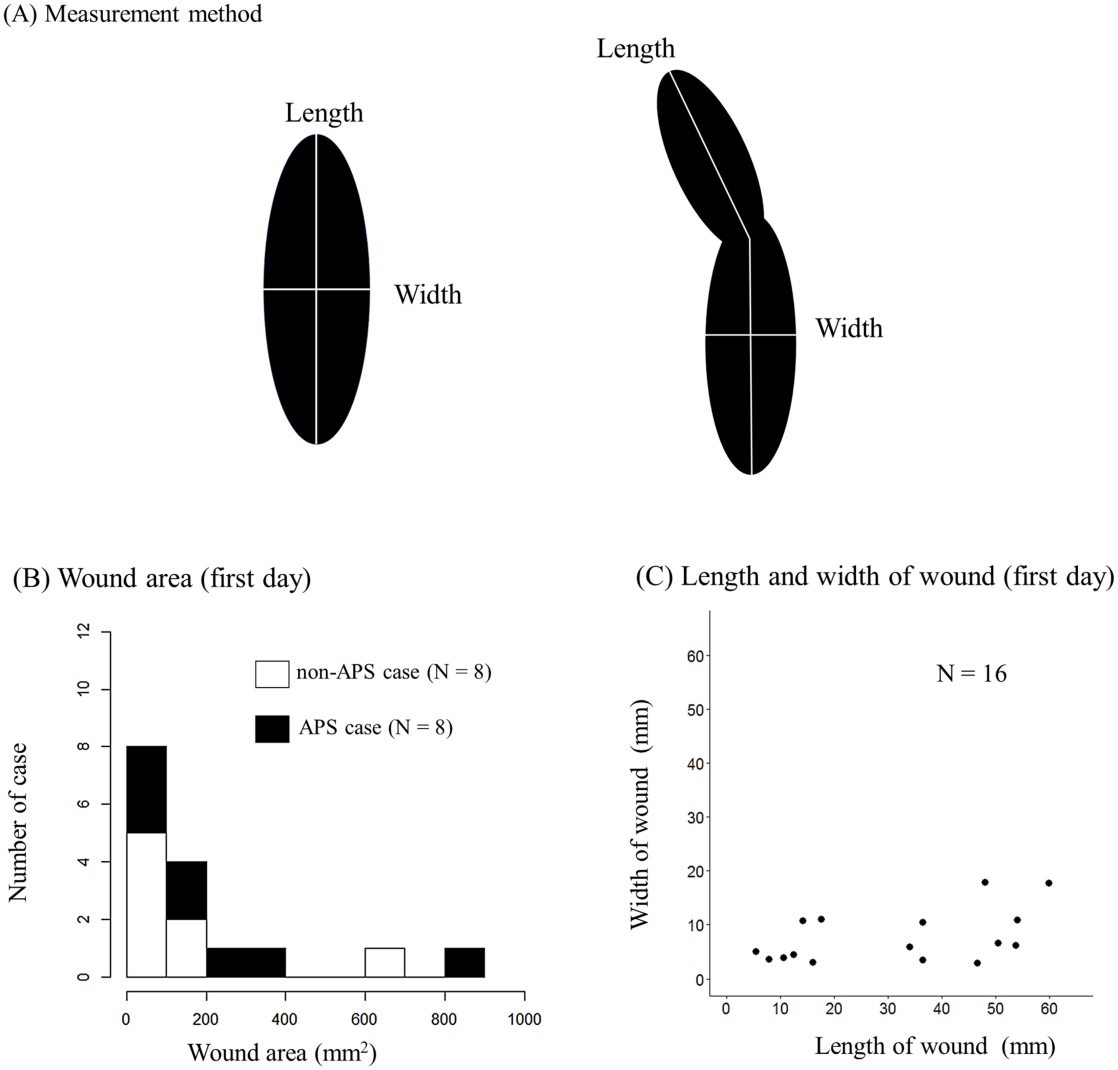
Wound area, length, and width. (A) Measurement of wound length and width. (B) Histogram of wound area on the first day (N = 16, S1 Table). Non- *Accidental peeling off scabs* (APS) cases are shown in white (N = 8), and APS cases are shown in black (N = 8). In the non-APS cases, the median wound area on the first day was 52.6 mm^2^ (range: 18.9–616.9 mm^2^) and the median healing time was 9.5 d (range: 6–27 d). Non-APS cases included five of the seven individuals. In eight APS cases, the median wound area on the first day was 165.3 mm^2^ (range: 25.0–812.3 mm^2^) and the median healing time was 18.5 d (range: 8–42 d). APS cases included five of the seven individuals. (C) Relationships between wound length and width on the first day of wounding (N = 16, S1 Table). The median length of wounds was 35.2 mm (N = 16, range: 5.5–60.0 mm) and the median width was 6.1 mm (N = 16, range: 2.9–17.9mm).

We used a video camera (Sony FDR-AX30) or digital camera (Sony Cyber-Shot HX DSC-HX60V) to record wounds (e.g., Fig 1). To measure the body size, we took photographs or videos of the subjects and natural scales (e.g., trees and stones) together. We captured the photograph when the natural scale and the target individual were in the same plane, parallel to the lens of the camera. After the subject had left, we placed a meter-stick and photographed the same location. After measuring the natural scale with the meter-stick, we measured the body parts of the subject (ear length, tail thickness, hand length, hand width, or wrist length) using ImageJ software (http://rsbweb.nih.gov/nih-image/). All male baboons except one were measured at least four times, and each body part of each male baboon was measured one to six times. The average size of each body part of each baboon was used as the scale for wound measurements. Wound length was measured as the longest span in any direction, and wound width as the longest span perpendicular to the length (Fig 2A). We followed the definition of wound area proposed by Rennert et al. [37]. We traced the wound edge in ImageJ using the computer mouse and calculated total wound area. Re-epithelialized tissue toward the wound edge was lighter in color than normal skin, and it was not included in the wound area measurement. When the cutaneous wound was considered as a single wound but not continuous wounds, we measured the healing time of the wounds separately. We measured the wound area twice to reduce measurement error and used average wound area in the analysis. The data are provided in S2 Table.

### Data analysis

#### Daily rate of cutaneous wounds

We determined the daily wound rate for each subject by using the following formula:

$$daily\;rate\;of\;cutaneous\;wounds=\frac{number\;of\;cutaneous\;wounds}{days\;during\;observation\;period\;of\;the\;subject,\;including\;non-observation\;days^{a}}$$

a: we counted the days during the observation period that the subject was checked in the AI group because we sometimes counted wounds that were not fresh and seemed to be a few days old.

#### Category of two successive measurement points in the wound area during healing process

In order to examine the healing delay due to APS, we divided the two successive measurement points of wound area during the healing process into three categories. (1) normal process (non-APS → non-APS), (2) during APS (non-APS → APS, or APS → APS), and (3) after APS (APS → non-APS). For the analysis, we used only the data when we could measure the wound area for two successive days.

#### Predicted delay time

The delay, in days, was predicted from the “healing model” we constructed based on the non-APS case by using generalized additive mixed effect models (GAMMs; see statistical analysis for details, Table 1 GAMM-1). The predicted delay was calculated using the following formula:

$$predicted\;delay\;time=(Pt_{1}-Pt_{0})+1$$

where t_0_: date of measurement, t_1_: the day after t_0_, W_0_: the wound area at t_0_, W_1_: the wound area at t_1,_ Pt_0_: the predicted healing day of W_0_ from the healing model, Pt_1_: the predicted healing day of W_1_ from the healing model.

**Table 1:** Coefficients of the generalized additive mixed effect models (GAMMs).

Models	Response variable	Error distribution	AIC	Explanatory variable	Estimate	SE	t	*P*	Difference between models (GAMM -1 vs. GAMM-2)
GAMM-1 non-linear model (8 cases, N = 43)	Number of days to healing	Gaussian	206.6	Intercept	5.43	0.44	12.25	< 0.01	*p* < 0.01 (X^*2*^ = 19.40, △df = 1)
				s (log_10_ [wound area])	a	-	-	< 0.01	
GAMM-2 linear model (8 cases, N = 43)	Number of days to healing	Gaussian	225.4	Intercept	-1.91	1.04	-1.84	0.07	
				log_10_ [wound area]	5.81	0.60	9.69	< 0.01	

When the size of wound area was larger, the number of days required for healing increased (GAMM-1, GAMM-2).

^a^: As the “wound area” is smoothed for modelling, this coefficient could not be evaluated.

The non-linear model (GAMM-1) was significantly different from the linear model (GAMM-2) and the Akaike Information Criterion (AIC) of the non-linear model was smaller than that of the linear model. Therefore, we adopted the non-linear model. The model residuals of GAMM-1 did not differ significantly from a normal distribution (Kolmogorov–Smirnov test, D = 0.19, *P* = 0.08). GAMM-1 was significantly better than the null model (X^2^ = 70.64, △d.f. = 2, *P* < 0.001).

The closer the value is to zero, the closer is the expected value. A positive value means that the healing time is longer than the expected value.

#### Proportion of peeled scabs in the wounded area

To assess the impact of the size of peeled scabs on healing delay, the proportion of peeled scabs in the wounded area was calculated as:

$$proportion\;of\;pealed\;scabs\;to\;the\;wound\;area\;\left(\%\right)=\frac{\left(area\;of\;scabs\;before\;the\;day\;of\;APS\right)-\left(area\;of\;scabs\;at\;the\;day\;of\;APS\right)}{\left(wound\;area\;before\;the\;day\;of\;APS\right)x100}$$

#### Statistical analysis

All tests were performed in R 3.4.2 [38], with packages including lme4 [39] and gamm4 [40]. To assess the effects of wound area on the number of days to healing, we used GAMMs, assuming a normal distribution, with “number of days to healing” as the response variable, and “log _10_ [wound area]” as the explanatory variable (Table 1, GAMM-1, GAMM-2). In addition, we set each case as a random effect. For this analysis, we used eight non-APS cases (Fig 2B, S2 Table). We removed the puncture wounds from our analysis, because these wounds were likely to be deep, and their depth could not be measured. The wound area, with the fractional part truncated, was log_10_-transformed. As a wound area of “0” cannot be log-transformed, a wound area of “0” was treated as “0” in this analysis (S2 Table). GAMMs provide a flexible, nonparametric form of regression modelling that uses smooth functions to model nonlinear relationships [40, 41]. In the non-linear model, the explanatory variable “log _10_ [wound area]” was fitted with a smooth function. To investigate whether the non-linear model (using the smooth function) was better than the linear model, these models were compared using a likelihood ratio test (ANOVA function with the argument test set to X²) and the Akaike Information Criterion (AIC). Generally, the model with a lower AIC value is assumed to be better fit [42]. We also compared the non-linear model with a null model (only with a random variable) by using a likelihood ratio test when we found significant effects (*P* < 0.05) of the explanatory variable using the Wald statistic in the model. After the model was constructed, we performed the Kolmogorov–Smirnov test to check the model for residual normality. Based on the results of these tests, we adopted the non-linear model (Table 1, GAMM-1).

To assess the influence of APS on healing delay, we used generalised linear mixed effect models (GLMMs), assuming a normal distribution, with “predicted delay time” as the response variable, and “category of two successive measurement points (normal process, during APS, after APS)” as the explanatory variable. In addition, we set each case and individual ID as random effects. We used eight APS cases (Fig 2B, S2 Table) in this analysis. Significance of the explanatory variable was tested using the Wald statistic. Similarly, we compared the model with a null model (only with a random variable) by using a likelihood ratio test when we found significant effects of the explanatory variable in the model. After the model was constructed, we performed the Kolmogorov–Smirnov test to check the model for residual normality.

## Results

### Rates of cutaneous wounds and wound healing time

We recorded 61 cutaneous wounds (puncture: 3 cases, cut and tear: 54 cases, and confirmation of bleeding only: 4 cases) and no individuals died due to cutaneous wounds. The daily rate of wound in the adult males in our study was 0.032 ± 0.016 cutaneous wounds per male per day (mean ± SD, N = 7, range = 0.003–0.047, i.e., 1 wound every 31 d, S3 Table).

In 16 cases, we were able to assess the number of days required for the wound to heal. Of these 16 wounds, the median wound area on the first day was 84.3 mm^2^ (range: 18.9–812.3 mm^2^) and the median healing time was 13 d (range: 6–42 d). No significant correlation was noted between the length and width of wounds (Pearson correlation, 0.47; *P* = 0.63, N = 16; Fig 2C).

**Fig 3:**
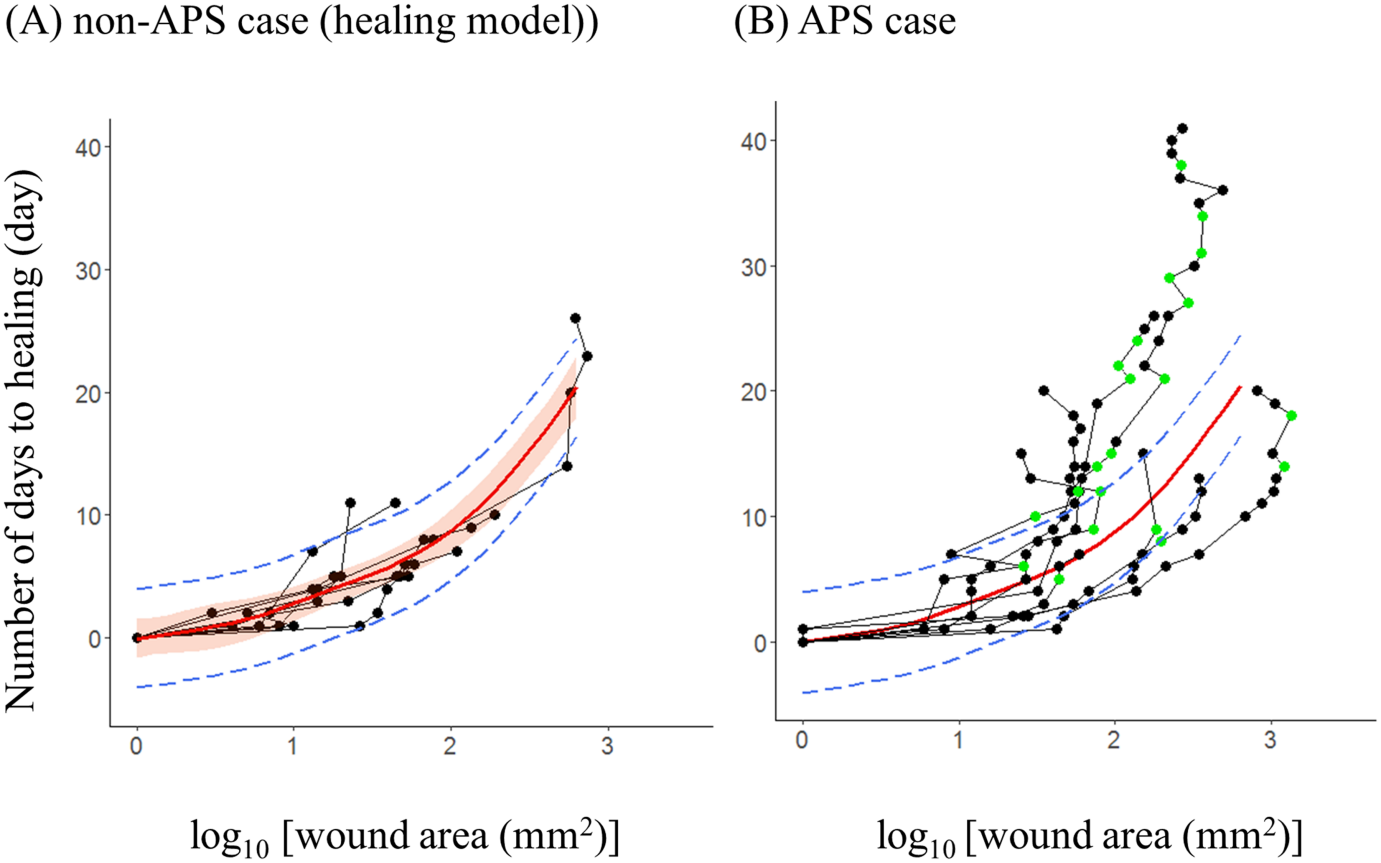
Relationship between wound area and healing time. (A) Eight non- *Accidental peeling off of scabs* (APS) cases (N = 43, S2 Table). The healing curve for each case (black dots and lines), regression curve (bold red lines, Table 1 GAMM-1), and 95% confidence interval (red-shaded area) are shown. The prediction interval (blue dashed lines) is the predicted value ± (SD of model residuals) × 2. We showed the same regression curve and prediction interval is shown in (B)., (B) Eight APS cases (N = 104, S2 Table). Green dots indicate the point at which the scab was peeled off (APS). In five of eight APS cases, multiple measuring days showed APS during the healing process.

Because eight of the 16 cases included the day of APS, we determined the influence of wound area on the number of days to healing by using the eight non-APS cases. When the wound area was larger, the number of days required for healing increased (GAMM, *P* < 0.01; Fig 3A, Table 1 GAMM-1; hereafter, this model is referred to as the “healing model”). No inflection points were found in the smoothing spline.

### Detail of APS cases

**Fig 4:**
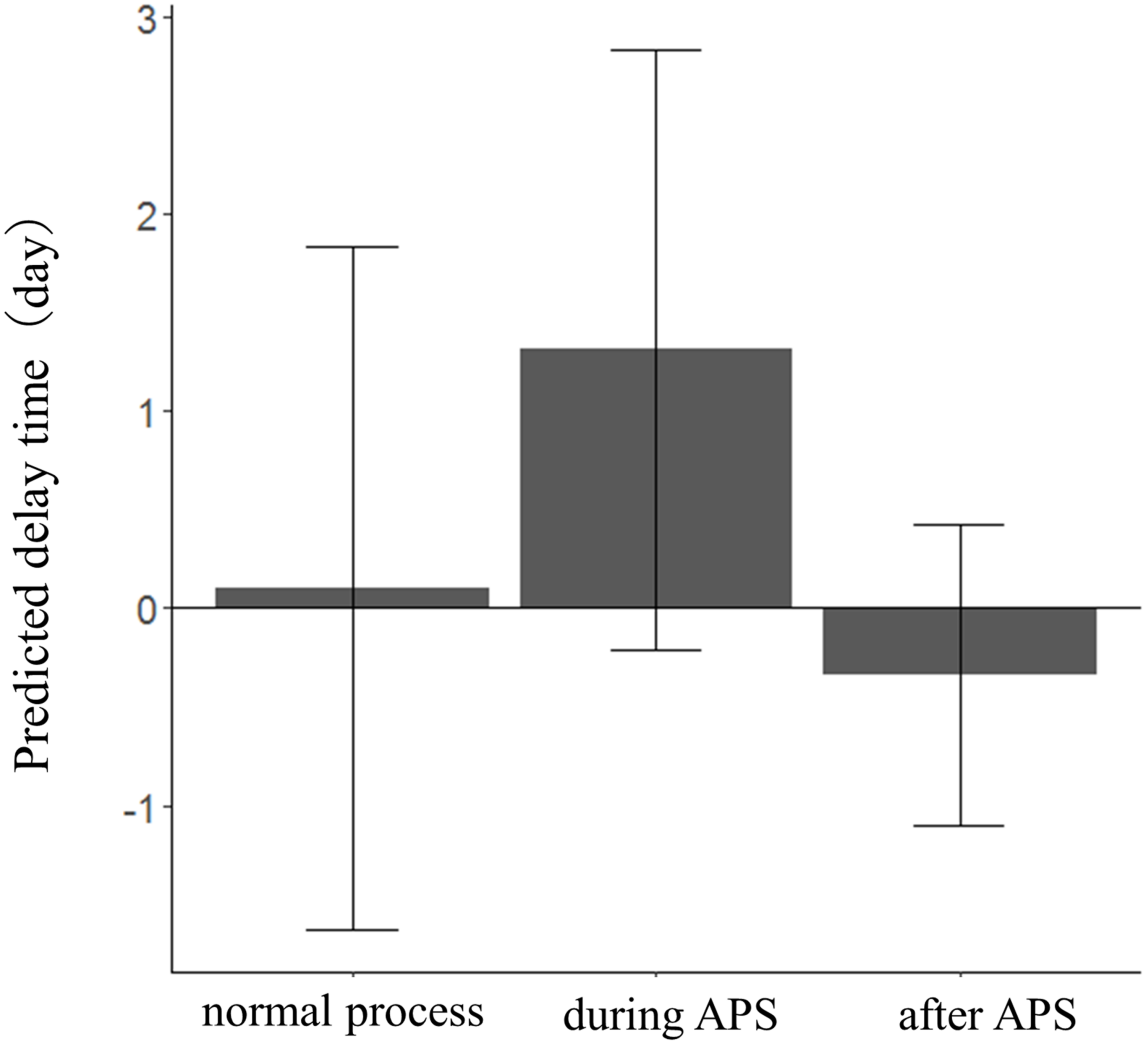
Predicted delay time and *Accidental peeling off of scabs* (APS). The bar and vertical line on the bar graph, represent the mean ± SD of the data in each category.

Based on the eight APS cases (Fig 3B), we calculated the predicted delay time. The mean proportion of peeled scabs to the wound area was 58% (N = 16, range = 4–100%, S4 Table), and no significant correlation was noted between the predicted delay time and the proportion of pealed scab in the wound area (Pearson correlation, -0.15; *P* = 0.59, N = 16, S4 Table). The value of the predicted delay time of “during APS” was larger than that for "normal process" (Fig 4, Table 2). This suggests that healing time became longer when the scab was peeled off. On the other hand, no difference was found between “normal process” and “after APS”. In four of the eight APS cases, we observed that the individuals touched their wound area, and picked up the scab using their fingers.

**Table 2:** Coefficients of the generalized linear mixed effect models (GLMMs).

Error distribution	Response variable	Explanatory variable		Estimate	SE	t	*P*
Gaussian	Predicted delay time (day) (N = 61)	Intercept		0.11	0.26	0.41	0.68
		Category of two successive measurement points	Normal process	0	0	-	-
			During APS	1.21	0.47	2.60	< 0.05
			After APS	-0.44	0.61	-0.73	0.47

The model residuals did not differ significantly from a normal distribution (Kolmogorov–Smirnov test, D = 0.12, *P* = 0.33). This model was significantly better than the null model (X^2^ = 8.07, △d.f. = 2, *P* < 0.05).

## Discussion

In our wild baboons study, we showed that the individual wound rate was one per month on average, and none of our study baboons died as a consequence of their wounds. In 16 cases, the median healing time was 13 d. When the scab was peeled off accidentally (APS), healing time becomes longer. In addition, one of the causes of APS was scab-picking behavior in wild baboons.

### Healing model and the influence of APS on healing time

Healing time can be predicted from the size of the wound area in terms of normal healing process of cutaneous wounds: scabs were formed on the wound area after wounding, and the healing was finished as scabs was peeled off naturally [20]. In a previous study, Drews [10] examined the relationships between healing time and wound length, and suggested that wound length is an insufficient indicator of healing time in yellow baboons *(Papio cynocephalus*) [10]. Measuring not only wound length [10, 17, 18], but also wound area is preferential to estimate healing time because wound length and width are not correlated.

Wound healing time was affected by APS as it was longer “during APS” than in “normal process”. Although internal factors (hormones and reproductive state) have been proposed to delay wound healing [14, 15, 17, 18], external factors (e.g., APS) also need to be taken into consideration. Secondary damage of wounds (i.e., APS) is not rare in wild baboons, as five of the seven individuals observed experienced APS once during our 11-month study period. Based on the “healing model” developed here, we showed that not only the healing time but also the delay time caused by external factors (e.g., APS) can be estimated.

The secondary damage during healing process resulted in delayed healing. However, the proportion of pealed scabs in the wounded area was not correlated with healing delayed. During APS, healing time might be delayed when the tissue is damaged, or the wound area expands. After APS, no difference in the delay time was found between “normal process” and “after APS”. Although scabs have functions as barriers against foreign material [21], we did not observe any infection after APS. Thus, our result suggests that the influence of peeling scab on healing time and infection was not long and serious. After APS, we can re-predict healing time from wound area using the “healing model.”

One of the causes of APS in wild baboons was scab-picking, and baboons worsened their own wounds. In humans, skin-picking behaviour, including APS [43] is considered a “self-injurious behaviour” (SIB) that is defined as a physical attack on or potentially damaging manipulation of one’s body [44], and the baboons’ scab-picking behaviour may be included in SIB. The skin-picking behavior has been reported to relieve tension in humans [43, 45]. In captive non-human primates, SIB has the same effect as in humans, and it is associated with heart rate return to baseline exposed to stressors [46]. In many instances, self-directed biting has been reported not to result in actual wounds [46, 47], and the healing cost of SIB seems to be low in primates. Our results showed that the cost of APS in terms of healing time was on mean 1.3 days (Fig 4). Thus, the healing cost of APS was not high compared with that of acquiring a new wound. Wounded male anubis baboons generally avoid interactions with other baboons [11, 48] which might avoid additional wounding due to further male attacks. After wounding, baboons sometimes may peel their scabs for their tension relief.

### Impact of wounds on wild baboons survival

Wounds had little effect on the survival of male baboons during our study periods and the observed wounds seemed not serious. We observed one wound per month on average, and the median healing time was 9.5 d (non-APS cases) or 18.5 d (APS cases). Previous studies on wild yellow baboons, reported one per 1.5 month [10] and the median healing time of the wounds was 26–30 d [10, 17]. In previous studies [10, 17], the confirmation interval of wounds was 6 to 14 d, which is longer than that in our study. Thus, recording small wounds (e.g., healing time is within one week) would have been difficult in previous studies [10, 17]. In addition, APS was not recorded in previous studies [10, 17]. Therefore, accurate comparison with previous studies is difficult. However, we can suggest that half of the wounds are assumed to heal within a month in wild male baboons. Cutaneous wounds that take more than one month to heal can be regarded as relatively severe wounds for wild baboons.

Behavioral mechanisms that prevent aggressive escalation are thought to be selected in social animals [10, 33, 49, 50]. In this study, most of cutaneous wounds were mild wounds (healing time was less than two weeks). In anubis baboons, approximately 50% of aggressive behaviors include chasing [34], in which attack recipients can avoid serious wounds by evading attacks or escaping. In mammals, lethal physical wounds occur when the costs of losing a mating contest are high or when escaping from attackers is difficult (e.g., coalitional attacks) [23], and lethal injuries have been reported in anubis baboons in the situations described above [11, 48]. When recipients do not or cannot escape from attackers, they might receive serious wounds. Lethal physical attacks have been widely reported in social mammals [23, 24]. In the future, clarifying the patterns of serious physical attacks in social mammals (e.g., primates) might be possible by measuring the severity of wounds (e.g., healing time). Moreover, we generated a model to predict healing time based on wound area that can be used in future comparative research. To our knowledge, this is the first study to measure wound area continuously, from the first day of wounding to the day of healing, not only in non-human primates, but also in large- and medium-sized wild animals. The healing time of studied animals will be based on initial pilot studies that document the comprehensive healing profile [1], such as our study.

## Supporting information

S1 Table Data on the wound area, length, and width in the day of wounding (first day). (XLSX)

S2 Table Data on the wound area and healing time. (XLSX)

S3 Table Data on the daily rate of wound. (XLSX)

S4 Table Data on proportion of peeled scabs in the wounded area. (XLSX)
